# Pediatric Urinary Retention and Constipation: Vaginal Agenesis with Hematometrocolpos

**DOI:** 10.5811/westjem.2015.1.25384

**Published:** 2015-03-17

**Authors:** Rebekah Heckmann, Francisco Alexander de la Fuente, Jason D. Heiner

**Affiliations:** * University of Washington, Division of Emergency Medicine, Seattle, Washington; † PeaceHealth Peace Island Medical Center, Department of Emergency Medicine, Friday Harbor, Washington

An 11-year-old healthy female presented to the emergency department with three days of worsening suprapubic pain, urinary retention, and constipation. She was afebrile with normal vital signs. Her physical examination was notable for suprapubic distention and bulging pink vaginal tissue at the introitus. Bedside ultrasound suggested a distended bladder. Placement of a Foley catheter returned 550mL of urine with improvement of the patient’s discomfort, but repeat ultrasound visualized a persistent hypoechoic mass adjacent to the newly decompressed bladder (Figure). The obstructive cause of her abdominal pain and urinary retention was revealed by magnetic resonance imaging (MRI) of the pelvis, which confirmed distal vaginal agenesis with uterine distention from hematometrocolpos (Figure). A Foley catheter was temporarily left in place, and after pediatric and gynecological consultation and operative intervention, she was later free of obstructive symptoms after surgical correction of her vaginal agenesis and hematometrocolpos.

Müllerian duct abnormalities, such as imperforate hymen, transverse vaginal septum, and vaginal agenesis, may be associated with abdominal pain or other symptoms of pelvic outlet obstruction, hematocolpos, and amenorrhea in the early adolescent years.^1^–^4^ While the prevalence of congenital uterine anomalies is estimated at 6.7%, Müllerian agenesis with lack of vaginal or uterine development is thought to only occur in one out of every 4,000–10,000 females.^1^,^2^ These errors in development are strongly associated with a number of other congenital anomalies including urinary tract abnormalities such as renal agenesis in an estimated 18–40% of patients, particularly when a hymen is absent.^3^–^5^ Visualization of vaginal-appearing tissue on physical examination instead of bulging bluish tissue more indicative of an imperforate hymen may suggest vaginal agenesis, but both ultrasound and MRI are recommended to adequately characterize pelvic and neighboring anatomy.^6^

## Figures and Tables

**Figure:**
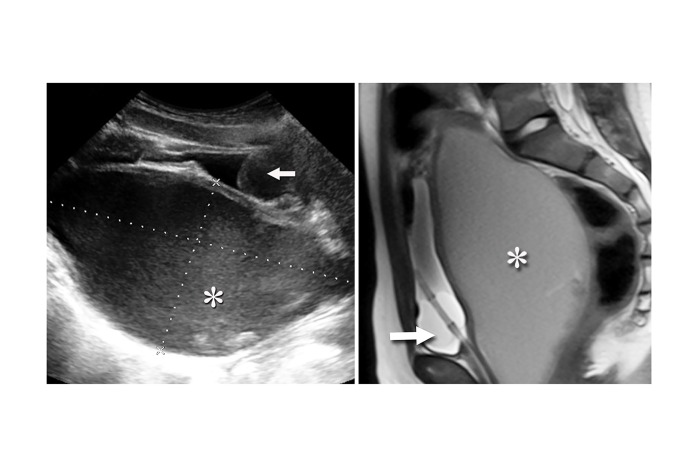
Long axis transabdominal sonographic view (left) of the patient’s abdomen revealing intrauterine low-level echogenic material (asterisk) communicating with the vaginal vault and a Foley catheter within a decompressed bladder (arrow). Sagittal magnetic resonance image (right) demonstrating fluid-filled distention (asterisk) of the patient’s uterus and vagina to the level of the introitus and a Foley catheter within the decompressed bladder (arrow).
